# Exploration of the *Ixodes ricinus* virosphere unveils an extensive virus diversity including novel coltiviruses and other reoviruses

**DOI:** 10.1093/ve/veab066

**Published:** 2021-07-12

**Authors:** Bert Vanmechelen, Michelle Merino, Valentijn Vergote, Lies Laenen, Marijn Thijssen, Joan Martí-Carreras, Edwin Claerebout, Piet Maes

**Affiliations:** Department of Microbiology, Immunology and Transplantation, Laboratory of Clinical and Epidemiological Virology, Rega Institute for Medical Research, KU Leuven—University of Leuven, Herestraat 49, Box 1040, Leuven BE3000, Belgium; Department of Microbiology, Immunology and Transplantation, Laboratory of Clinical and Epidemiological Virology, Rega Institute for Medical Research, KU Leuven—University of Leuven, Herestraat 49, Box 1040, Leuven BE3000, Belgium; Department of Microbiology, Immunology and Transplantation, Laboratory of Clinical and Epidemiological Virology, Rega Institute for Medical Research, KU Leuven—University of Leuven, Herestraat 49, Box 1040, Leuven BE3000, Belgium; Department of Microbiology, Immunology and Transplantation, Laboratory of Clinical and Epidemiological Virology, Rega Institute for Medical Research, KU Leuven—University of Leuven, Herestraat 49, Box 1040, Leuven BE3000, Belgium; Department of Microbiology, Immunology and Transplantation, Laboratory of Clinical and Epidemiological Virology, Rega Institute for Medical Research, KU Leuven—University of Leuven, Herestraat 49, Box 1040, Leuven BE3000, Belgium; Department of Microbiology, Immunology and Transplantation, Laboratory of Clinical and Epidemiological Virology, Rega Institute for Medical Research, KU Leuven—University of Leuven, Herestraat 49, Box 1040, Leuven BE3000, Belgium; Faculty of Veterinary Medicine, Laboratory of Parasitology, Ghent University, Salisburylaan 133-D13, Merelbeke BE9820, Belgium; Department of Microbiology, Immunology and Transplantation, Laboratory of Clinical and Epidemiological Virology, Rega Institute for Medical Research, KU Leuven—University of Leuven, Herestraat 49, Box 1040, Leuven BE3000, Belgium

**Keywords:** *Ixodes ricinus*, tick, virus discovery, Eyach virus, *Coltivirus*, reovirus, Belgium

## Abstract

Recent metagenomics studies have revealed several tick species to host a variety of previously undiscovered RNA viruses. *Ixodes ricinus*, which is known to be a vector for many viral, bacterial, and protozoan pathogens, is the most prevalent tick species in Europe. For this study, we decided to investigate the virosphere of Belgian *I. ricinus* ticks. High-throughput sequencing of tick pools collected from six different sampling sites revealed the presence of viruses belonging to many different viral orders and families, including *Mononegavirales, Bunyavirales*, *Partitiviridae*, and *Reoviridae*. Of particular interest was the detection of several new reoviruses, two of which cluster together with members of the genus *Coltivirus*. This includes a new strain of Eyach virus, a known causative agent of tick-borne encephalitis. All genome segments of this new strain are highly similar to those of previously published Eyach virus genomes, except for the fourth segment, encoding VP4, which is markedly more dissimilar, potentially indicating the occurrence of a genetic reassortment. Further polymerase chain reaction–based screening of over 230 tick pools for 14 selected viruses showed that most viruses could be found in all six sampling sites, indicating the wide spread of these viruses throughout the Belgian tick population. Taken together, these results illustrate the role of ticks as important virus reservoirs, highlighting the need for adequate tick control measures.

## 1. Introduction

Recent metagenomics studies have revealed the extensive virus diversity within arthropods (Li et al., 2015; Shi et al., 2016). Of particular interest amongst arthropods are the haematophagous vectors. These animals feed on the blood of vertebrate hosts, presenting the ideal conditions for the transmission of pathogens. In addition to mosquitoes, ticks are some of the most important vectors of infectious diseases, in humans, livestock, and wildlife animals (Mansfield et al., 2017). Ticks are known to carry a diverse range of pathogens, including protozoa, bacteria, helminths, and viruses, although in Europe they are predominantly known for their ability to transmit *Borrelia burgdorferi* bacteria, the causative agent for Lyme disease (Bush and Vazquez-Pertejo 2018; Boulanger et al., 2019; Madison-Antenucci et al., 2020). However, ticks also act as vectors for multiple viral pathogens, including tick-borne encephalitis virus (TBEV), the most prevalent viral tick-borne zoonotic agent in Europe, Crimean-Congo haemorrhagic fever virus, and Colorado tick fever virus (Shi et al., 2018). In the last decade, many articles have been published looking at the virus diversity in different tick species in different regions, revealing the presence of many previously undiscovered viruses (Tokarz et al., 2014, 2018; Xia et al., 2015; Moutailler et al., 2016; Pettersson et al., 2017; Harvey et al., 2019; Meng et al., 2019). Although estimating the relative risk of these novel viruses for human, animal, and plant health is difficult, their phylogenetic clustering within specific virus families and genera that are known to harbour tick-borne human, animal, and plant pathogens, such as *Reoviridae, Nairoviridae, Flaviviridae*, or *Phenuiviridae*, hints at the pathogenic potential for at least some of them.

Ticks are divided into three families: the *Argasidae* or ‘soft ticks’, the *Ixodidae* or ‘hard ticks’, and the *Nuttalliellidae*. Unlike the latter, which contains only one species, the families *Argasidae* and *Ixodidae* harbour several hundred member species, split across multiple genera (Guglielmone et al., 2010). In Belgium, only a limited number of tick species have been described, and almost all reported tick bites can be attributed to the castor bean tick or *Ixodes ricinus*, the type species of the genus *Ixodes* in the family of ‘hard ticks’ (Obsomer et al., 2013; Lernout et al., 2019). This predominantly European tick species requires three blood meals during its life cycle: to moult from larva to nymph and from nymph to adult, and to lay eggs once it has reached adulthood (Walker 2019). This triple-host life cycle makes *I. ricinus* an important vector for the transmission of several tick-borne diseases between a variety of mammalian hosts, including humans. Several viruses that pose a threat to human and animal health are already known to be transmitted by *I. ricinus* ticks, although, with the exception of TBEV, studies concerning human diseases of viral origin spread by *I. ricinus* are limited (see Hubalek and Rudolf, 2012, for a detailed overview). Furthermore, the ongoing discovery of new (putative) viral pathogens, such as the newly described Alongshan virus, which was recently reported to be present in European *I. ricinus* ticks, indicates that *I. ricinus* ticks might harbour more zoonotic viruses than hitherto assumed (Kuivanen et al., 2019).

In recent years, a number of studies have delved deeper into the virus diversity in *I. ricinus* in Europe, revealing the presence of several previously undetected viruses (Moutailler et al., 2016; Pereira et al., 2017; Pettersson et al., 2017; Kuivanen et al., 2019; Ohlendorf et al., 2019). In 2017, we reported the discovery of a novel nairovirus, Grotenhout virus, in Belgian *I. ricinus*, which was identified through next-generation sequencing of a pool of adult ticks (Vanmechelen et al., 2017). Because we also detected traces of other viruses, we decided to sequence additional tick pools to further characterize the virus diversity in Belgian ticks. Here, we report the detection of several novel as well as previously described viruses, including a rhabdovirus and multiple reoviruses. Additionally, we performed polymerase chain reaction (PCR) screening for 14 different viruses, including the novel viruses reported here as well as some other viruses known to be carried by *I. ricinus*, on more than 200 tick pools from six sampling locations in Belgium, providing a broad overview of virus prevalence in Belgian *I. ricinus* ticks.

## 2. Materials and methods

### **2.** **1** Sample collection

**Figure 1.**
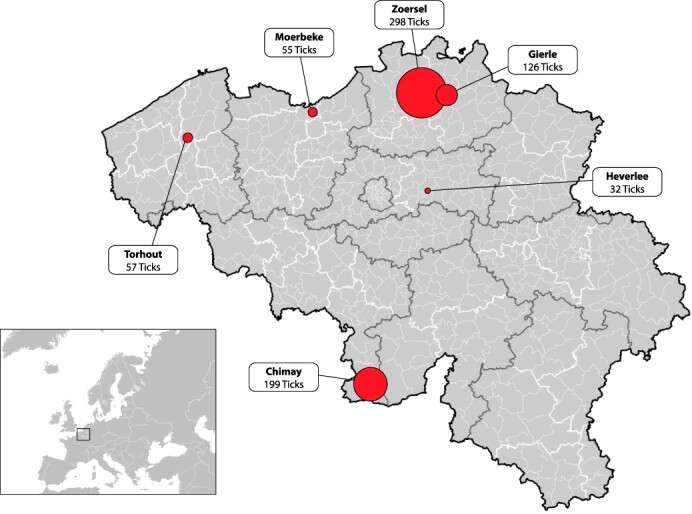
Tick sampling sites. Ticks were collected from six sampling sites in Belgium, marked in red and scaled according to the number of ticks caught at each location. Dark grey, white, and light grey lines represent province, arrondissement, and municipality borders, respectively.

Ticks were collected between 2009 and 2017 from six sampling locations in Belgium (Torhout [51°4ʹ10.5″N, 3°3ʹ25.9″E], Moerbeke [51°11ʹ39.5″N, 3°55ʹ4.3″E], Zoersel [51°14ʹ41.8″N, 4°40ʹ49″E], Gierle [51°17ʹ19.3″N 4°53ʹ8.2″E], Chimay [50°01ʹ35.2″N 4°14ʹ52.5″E], and Heverlee [50°51ʹ27.5″N 4°39ʹ56.4″E]), by flagging the low-growing vegetation using a 1-m-by-2-m white cotton flag. Ticks from Gierle, Chimay, and Heverlee were kept at −20°C for long-term storage, while ticks from Torhout, Moerbeke, and Zoersel were stored at room temperature in 100 per cent ethanol. A total of 767 ticks were collected, including 33 adults, 513 nymphs, and 221 larvae. All ticks were sorted based on their location of origin and their developmental stage, and all were identified as *I. ricinus* based on phenotypical characteristics. An overview of the different sampling locations and the number of ticks caught at each location can be found in Fig. 1.

### **2.** **2** RNA extraction

Each adult tick or pool (1–10) of nymphs/larvae (see Supplementary Table S1) was added to a tube containing 100 µl phosphate buffered saline and zirconium oxide beads and subsequently homogenized for 150 s at 4,000 g in a Bertin Minilys homogenizer. Total RNA was extracted from the homogenate using the RNeasy Mini Kit (QIAGEN, Leiden, Netherlands) according to the manufacturer’s instructions. DNA carry-over was further depleted by DNase treatment using 2U TurboDNase (Invitrogen, ThermoFisher Scientific, CA, USA), and the remaining RNA was re-purified using the RNeasy MinElute Cleanup Kit (QIAGEN, Leiden, Netherlands).

### **2.** **3** Attempt at virus isolation

Virus rescue was attempted using African green monkey kidney cells (Vero E6; American Type Culture Collection, C1008) and hamster kidney fibroblasts (BHK21). Vero E6 cells were maintained in Dulbecco’s Modified Eagle Medium (Thermo Fisher Scientific, MA, USA) supplemented with 10 per cent fetal bovine serum (FBS; Biowest, France), 1 per cent sodium bicarbonate, 1 per cent penicillin-streptomycin-l-glutamine, 1 per cent gentamicin, and 0.2 per cent fungizone (all Thermo Fisher Scientific). BHK21s were kept in Minimal Essential Medium Rega-3 (Thermo Fisher Scientific), supplemented with +10 per cent FBS and 200 mM l-glutamine (Thermo Fisher Scientific). To attempt virus rescue, cells were seeded in 6-well plates and allowed to grow overnight. The medium was removed from the resulting monolayers, which were subsequently inoculated with 200 µl fresh tick homogenate for 1 h, after which 2 ml 2 per cent FBS-supplemented medium was added. Cells were observed daily for cytopathogenic effect for 2 weeks, and the cell medium was refreshed every 3–4 days. Collected supernatant was subjected to RNA extraction using the RNeasy Mini Kit (QIAGEN), followed by reverse transcription PCR (RT-PCR) screening as described below.

### **2.** **4** Nanopore sequencing

RNA extracted from a single female adult tick was converted to complementary DNA (cDNA) using the Complete Whole Transcriptome Amplification Kit (WTA2, Sigma-Aldrich, Saint Louis, MO, USA). Purification of cDNA was done using the MSB Spin PCRapace kit (Stratec Molecular, Berlin, Germany). The resulting cDNA was prepared for nanopore sequencing using the SQK-LSK108 kit (Oxford Nanopore Technologies, Oxford, UK) and loaded on an R9.4.1 flow cell. Reads corresponding to tick ribosomal RNA (rRNA) were removed, and following size selection, reads between 200 and 2,000 nucleotides were assembled using Canu v1.5 (Koren et al., 2017). Identification of the resulting contigs was done using BLAST (https://blast.ncbi.nlm.nih.gov/Blast.cgi).

### **2.** **5** Illumina sequencing

Ten pools were made for Illumina sequencing, containing three to five RNA extracts each. An overview of the ten pools can be found in Table 1. RNA was converted to cDNA using the WTA2 Kit (Sigma-Aldrich). cDNA was purified using the MSB Spin PCRapace kit (Stratec Molecular). Sequencing libraries were prepared using the Illumina NexteraXT DNA Library Prep Kit (Illumina, San Diego, USA). Libraries were double indexed using Set A-barcoded adapters (Illumina, San Diego, USA). In-house modifications on the standard library preparation were conducted following Conceicao-Neto et al. (2018). Sequencing was performed on a HiSeq4000 platform (Illumina, San Diego, USA) at VIB Nucleomics Core (Leuven, Belgium). CLC Genomics Workbench (v10.1.1) was used to trim the reads by adapter content, quality (Phred q-score >35), and length (>25 bp) and to assemble the resulting trimmed reads (default settings). Diamond (v0.9.24) and KronaTools (v2.7.1) were used to classify the obtained contigs, using the blastx algorithm against the complete NCBI non-redundant protein database (nr; GenBank release 239) (Ondov, Bergman, and Phillippy 2011; Buchfink, Xie, and Huson 2015). The HMMER v3.3.2 web server (www.hmmer.org) was used to identify divergent virus segments that could not be classified by Diamond.

**Table 1.** Illumina sequencing data overview.

Pool number	RNA pools used^a^		#Ticks	Adult/nymph/larva	Location	#Reads	#Viral reads	#Contigs	#Viral contigs
S1	142 144	147 150	4	4 Adults	Moerbeke	62,886,446	977	4,874	13
S2	4 6	87 91	4	4 Adults	Chimay Gierle	65,898,424	1,576	6,918	16
S3	140 162	168	3	3 Adults	Heverlee Torhout	48,976,538	6,621	25,558	24
S4	143 146	149	3	3 Adults	Moerbeke	68,520,456	12,952	65,273	65
S5	89 90	95	3	3 Adults	Gierle	68,966,000	750,760	6,771	94
S6	129 159	161	7	3 Nymphs 4 Larvae	Heverlee Moerbeke	63,439,638	149,654	74,219	693
S7	47 48	51 56	12	12 Nymphs	Chimay	76,725,768	7,301	15,224	66
S8	107 110	114 126	12	12 Nymphs	Chimay Gierle	58,129,700	366,933	53,572	132
S9	106 110 111	112 113	15	15 Nymphs	Gierle	83,201,118	293,361	35,840	842
S10	39 44 45	135 139	13	13 Nymphs	Chimay Heverlee	70,097,012	47,741	15,718	96

a See Supplementary Table S1.

### **2.** **6** PCR screening and genome completion

The Qiagen OneStep RT-PCR kit was used to screen for the different viruses and to complete gaps in the obtained genome sequences. The following cycling conditions were used: 50°C for 30 min, 95°C for 15 min, 40 cycles of 94°C for 30 s, a primer-specific annealing temperature for 30 s and 72°C for 60 s, and a final extension step at 72°C for 10 min. Completion of genome ends was done using the Roche 5ʹ/3ʹ RACE kit, 2nd generation. An overview of all used screening primer sets and annealing temperatures can be found in Supplementary Table S2. PCR products were visualized by 2 per cent agarose gel electrophoresis and Sanger sequenced to confirm their identity. Sequencing was done by Macrogen Europe (Macrogen Europe B.V., Amsterdam, The Netherlands) after the products were purified in-house with PureIT ExoZAP (Ampliqon, Odense, Denmark).

### **2.** **7** Phylogenetic analysis

For the newly discovered rhabdovirus and reoviruses, multiple sequence alignments were made based on deduced amino acid sequences of the RNA-dependent RNA polymerase (RdRp) sequence of members of the families *Rhabdoviridae* and *Reoviridae*. For the rhabdovirus phylogenetic tree, amino acid sequences for all currently recognized International Committee on the Taxonomy of Viruses species, supplemented with all unclassified rhabdoviruses >5,500 bp (NCBI: txid35303), were downloaded from NCBI-Nucleotide (https://www.ncbi.nlm.nih.gov/nuccore). Seqkit was used to keep only protein sequences >1,800 amino acids, which were aligned using MAFFT (v7.310; localpair algorithm) (Katoh and Standley 2013; Shen et al., 2016). Based on this alignment, the closest related sequences to Chimay rhabdovirus (and Rabies virus as an outgroup) were realigned using MAFFT. The resulting alignment was trimmed using TrimAl (v1.4.rev15; gappyout setting), and model selection and phylogenetic tree calculation were done using IQ-TREE (v.1.6.12), employing 1,000 bootstrap replicates (Capella-Gutierrez, Silla-Martinez, and Gabaldon 2009; Nguyen et al., 2015). The resulting tree was visualized using Figtree v1.4.3. For the reovirus tree, the RdRp protein sequence was deduced from all (putative) members of the subfamily *Spinareovirinae* for whom an RdRp-encoding segment could be identified. Alignment generation, trimming and phylogenetic tree calculation, and visualization were performed as described above. An overview of the accession numbers of all used sequences can be found in Supplementary Table S4.

## 3. Results

### **3.** **1** Detection of multiple viruses in a single tick

**Figure 2.**
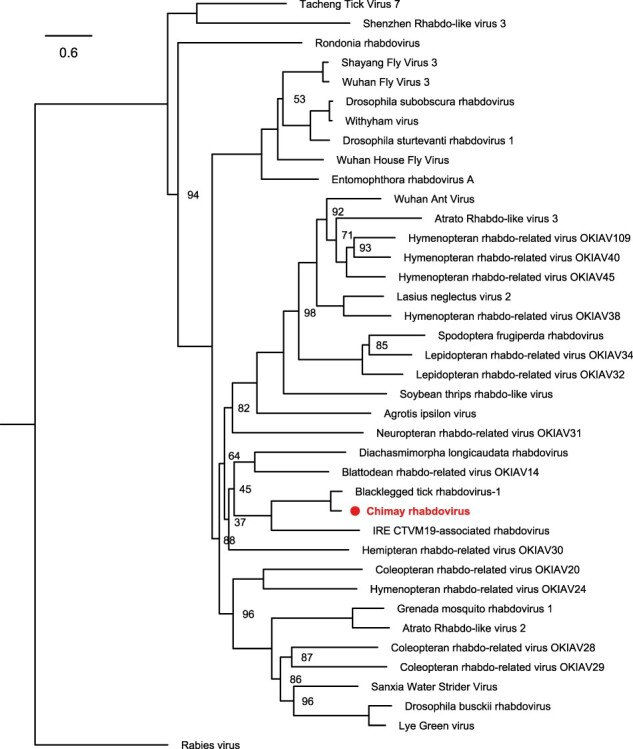
Phylogenetic clustering of Chimay rhabdovirus. Maximum-likelihood tree based on the protein sequence of the RdRp of Chimay rhabdovirus and the closest-related (putative) members of the family *Rhabdoviridae*, including Rabies virus as an outgroup. Chimay rhabdovirus clusters near Blacklegged tick rhabdovirus-1 and IRE CTVM19-associated rhabdovirus, both tick-derived viruses. The scale bar indicates the number of amino acids substitutions per site. The numbers at the node indicate the bootstrap support for each node, based on 1,000 replicates. Only values <100 are shown. All used accession numbers are provided in Supplementary Table S4.

We reported previously the discovery of a novel nairovirus, Grotenhout virus, in a pooled library of ten adult tick RNA extracts (Vanmechelen et al., 2017). Even though we recovered a complete L and S segment from the Illumina data, we found no traces of an M segment. However, we did find one 267-bp contig that resembled part of a phlebovirus segment. In an attempt to obtain more sequence information for this novel phlebovirus and to simultaneously look for the missing Grotenhout virus M segment, we screened all ten RNA extracts used to make the Illumina pool described in Vanmechelen et al. (2017) by RT-PCR for Grotenhout virus and the novel phlebovirus and selected one dual-positive extract (see Supplementary Table S1, extract 7) for RNA sequencing using the Oxford Nanopore MinION. This run yielded ∼4.6 million reads, covering ∼1.81 Gb. Following rRNA removal and length trimming (keeping reads between 200 and 2,000 bp), assembly using Canu (v1.5) yielded 31 contigs. Together, these contigs cover the full length of both the Grotenhout virus L and S segments as well as the L and S segments of the aforementioned phlebovirus, which we provisionally named Leuven phlebovirus (GenBank ID: MG407659-MG407660). Genome sequences similar to this virus (98.5–99.1 per cent nucleotide identity) have recently also been discovered in northern European ticks, under the name Norway phlebovirus 1 (Pettersson et al., 2017). All four segments had excellent coverage (see Table 2), but for neither virus, a contig matching a putative M segment was detected. The remaining contigs could all be classified as being of host or bacterial origin, except for three contigs that displayed limited similarity with members of the family *Rhabdoviridae* and appeared to be part of the same novel rhabdovirus. Correction by Sanger sequencing and RACE was used to obtain the complete 13,706-bp genome sequence for this virus, which was given the name Chimay rhabdovirus (GenBank ID: MF975531). The genome has the typical rhabdovirus genome architecture, encoding five open reading frames (ORFs): 3ʹ-N-P-M-G-L-5ʹ and clusters alongside Blacklegged tick rhabdovirus 1 and IRE CTVM19–associated rhabdovirus, both tick-derived viruses (Fig. 2). Chimay rhabdovirus was recently classified as a separate species (*Chimay betaricinrhavirus*) in a novel genus (*Betaricinrhavirus*) in the family *Rhabdoviridae*.

**Table 2.** Tick extract 7 nanopore data.

Virus ID	Segment length (bp)	#Contigs	Merged contig length (bp)	#Reads	Average coverage
Grotenhout virus L segment	14,854	6	14,806	38,615	1,433X
Grotenhout virus S segment	3,728	3	3,716	27,061	3,564X
Leuven phlebovirus virus L segment	6,730	1	6,699	6,769	580X
Leuven phlebovirus virus S segment	2,501	1	2,467	9,816	1,806
Chimay rhabdovirus	13,706	3	13,562	3,210	125X

### **3.** **2** Tick sample set

**Figure 3.**
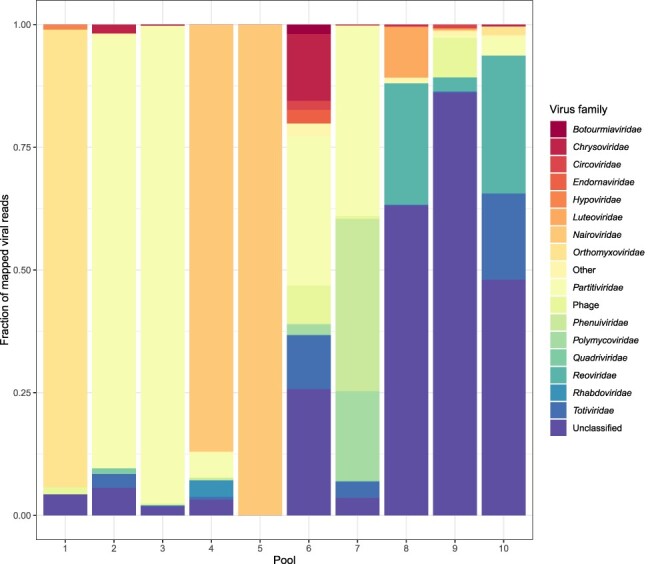
Abundance of viral families in the different Illumina pools. Fraction of mapped viral reads per pool, colour-coded based on the taxonomical classification (according to KronaTools) of the closest available reference, as determined by DIAMOND. Virus families representing <1 per cent of the virus reads in a sample are grouped as ‘Other’. Virus families known to be families of bacteriophages are grouped together as ‘Phage’, as they are unlikely to represent true tick-associated viruses.

The finding of three separate viruses in the RNA isolated from a single tick prompted us to further investigate the viral diversity in Belgian ticks. Therefore, we decided to collect additional ticks of different developmental stages and from different locations in Belgium and used next-generation sequencing for the detection of known and novel viruses in these ticks. Ten tick pools, made from pooling three to five RNA extracts, were sequenced on an Illumina 4000 HiSeq, yielding 48,976,538–83,201,118 reads per pool. Variable numbers of ticks from mixed locations of origin were used in the different pools to maximize the overall observed diversity. However, this approach also means that the pools should not be compared quantitatively with each other, as such comparisons would be biased due to the differences in sample handling and pooling. Following adapter trimming, between 4,874 and 74,219 contigs could be assembled for each pool (Table 1). Although the fraction of viral reads compared to host material was low for most pools, we did detect reads and contigs of many different viruses, with strong heterogeneity between pools (Fig. 3). While some pools were largely dominated by the presence of one specific virus (Pool 5), others displayed strong diversity, containing contigs of viruses belonging to many different families. Interestingly, while most of the virus families identified in our data are known to contain tick-borne viruses, we also observed fungus/plant-specific viral families (most notably *Botourmiaviridae, Chrysoviridae, Endornaviridae, Hypoviridae, Polymycoviridae*, and *Quadriviridae*) especially in Pool 6. This pool also displayed the largest diversity, but this is most likely attributable to the presence of fungi in the sample used for RNA extraction. Whether these fungi were infecting the ticks in this sample or should be considered environmental contaminants remains to be determined.

### **3.** **3** Reconstruction of viral genomes

**Figure 4.**
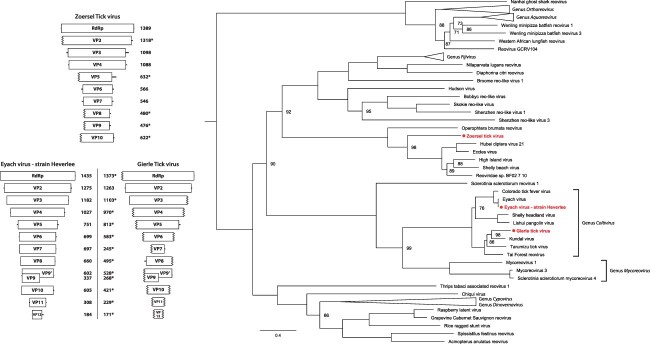
Reovirus phylogeny and genome organization. Left: genome organization of Eyach virus strain Heverlee, Gierle tick virus, and Zoersel tick virus, with segment lengths drawn according to scale. Ribbed lines indicate incomplete genome ends. The deduced amino acid length of all proteins is shown next to the corresponding segments. *Incomplete ORFs. Right: maximum-likelihood tree based on the protein sequence of the RdRp of all the (putative) members of the subfamily *Spinareovirinae* for whom such a sequence is available, shows Eyach virus strain Heverlee and Gierle tick virus clustering within the genus *Coltivirus*, with Gierle tick virus representing a novel species. Zoersel tick virus forms a separate branch within the subfamily, alongside several yet unclassified viruses. The scale bar indicates the number of amino acids substitutions per site. The numbers at the node indicate the bootstrap support for each node, based on 1,000 replicates. Only values <100 are shown. All used accession numbers are provided in Supplementary Table S4.

While for most detected viruses only fragments of the genome were retrieved, some were present more abundantly, allowing the reconstruction of near-complete genome sequences. These include Grotenhout virus and the aforementioned Leuven phlebovirus, which have also recently been observed in *I. ricinus* in Norway (Pettersson et al., 2017). With the exception of Norway luteo-like virus 2, all viruses that were discovered in this Norwegian study are also present in our data, although only Bronnoya virus, Norway partiti-like virus 1 and Norway luteo-like virus 1 had sufficient read support to recover contigs covering most of the genome. These contigs showed high nucleotide identity (>90 per cent) to the genomes of the Norwegian strains and are not further discussed here. Lastly, we also managed to retrieve near-complete genome sequences of three reoviruses. These include a novel strain of Eyach virus, named strain Heverlee (GenBank ID: MW874081-MW874092), as well as two previously undescribed viruses, named Gierle tick virus (GenBank ID: MW874093-MW874104) and Zoersel tick virus (GenBank ID: MW874105-MW874114), respectively. For Eyach virus strain Heverlee, a combination of Illumina and Sanger sequencing was used to obtain the complete sequence of all 12 genome segments. While almost all segments are highly similar (>94 per cent nucleotide identity) to previously identified strains of Eyach virus (Table 3), the fourth segment, encoding VP4, is markedly dissimilar, displaying <78 per cent nucleotide similarity. No other reovirus segments were detected in samples positive for this strain of Eyach virus. Akin to the Eyach virus, we managed to identify all 12 genome segments of Gierle tick virus, a putative new viral species in the genus *Coltivirus* most similar to Tarumizu tick virus and Kundal virus (Fig. 4). Lastly, we also managed to retrieve the near-complete genome of a third reovirus, Zoersel tick virus. Although markedly dissimilar from all currently classified reoviruses, this virus shares some similarity with the unclassified reoviruses High Island virus, Eccles virus, and ‘Reoviridae sp. BF02/7/10’, and the unclassified Riboviria Shelly beach virus and Hubei diptera virus 21. Together, these five viruses form a separate clade within the family *Reoviridae*, and while reoviruses typically have ten to twelve genome segments, only six genome segments are known for all five of these viruses. For Zoersel tick virus, we initially also identified only six genome segments using BLAST-based approaches. However, by reducing the stringency of our significance thresholds and by verifying putative hits with profile hidden Markov models using HMMER v3.3.2, additional three segments could be identified that shared similarity with Operophtera brumata reovirus, a ten-segmented, unclassified reovirus that phylogenetically clusters as a sister clade to the aforementioned six-segmented viruses (Graham et al., 2006; Potter et al., 2018). By looking through the unannotated contigs of our Illumina data, a putative tenth segment was identified for Zoersel tick virus. This contig shows no significant sequence or structural similarity to any known sequence (viral or otherwise) but was present in both Illumina pools containing Zoersel tick virus (and only these pools) and was the only unannotated contig in both sequencing pools that had comparable read coverage to the contigs of the nine segments that could be annotated (Supplementary Table S3). Its length also corresponds to the presumed length of the missing segment, and the sequence is predicted to encode one large ORF, as is characteristic for reovirus segments. Therefore, Zoersel tick virus is believed to have ten genome segments, akin to the distantly related Operophtera brumata reovirus. Attempts to isolate this virus (and Grotenhout virus and Leuven phlebovirus) from fresh tick homogenate on Vero E6 or BHK21 cells were unsuccessful. In addition to the three reoviruses for which (near-)complete genome could be retrieved, we also detected a fourth reovirus in one of the pools: Torhout tick virus (GenBank ID: MW874115-MW874117), a colti-like virus distantly related to the recently discovered Fennes virus, but only three partial segments could be reconstructed for this virus.

**Table 3.** Comparison of Eyach virus (RefSeq) and Eyach virus strain Heverlee.

Segment	Encoded protein	Segment length		Nucleotide identity (%)	Amino acid identity (%)
		Eyach virus	Heverlee tick virus		
1	VP1 (RdRp)	4,349	4,349	99.13	99.30
2	VP2	3,934	3,934	95.02	98.51
3	VP3	3,585	3,585	99.25	99.66
4	VP4	3,156	3,156	**77.96**	**88.12**
5	VP5	2,398	2,398	94.75	97.87
6	VP6	2,178	2,178	94.44	96.14
7	VP7	2,139	2,139	95.65	96.70
8	VP8	2,028	2,028	97.49	98.48
9	VP9	1,884	1,884	97.19	99.17
	VP9				99.41
10	VP10	1,879	1,879	94.89	97.02
11	VP11	1,002	1,002	99.40	99.35
12	VP12	678	678	95.87	94.57

The notably lower identity values for segment 4 are marked in bold.

### **3.** **4** Prevalence of selected viruses

**Figure 5.**
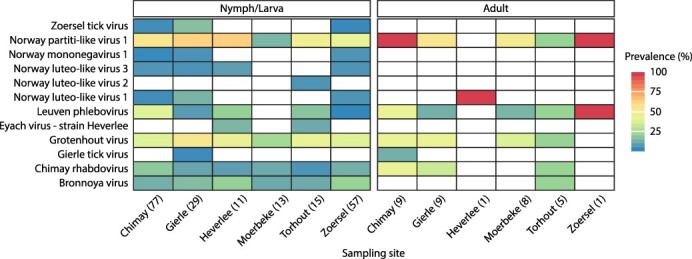
Virus prevalence at the different sampling sites. Overview of all PCR screening results shows that many of the detected viruses are found in different developmental stages and locations of origin. TBEV and Norway luteo-like virus 4 were not detected by PCR in any of the screened pools. Percentages shown here are likely over- or under-representations of the true values due to pooling of ticks (1–10 for nymphs/larvae) during extraction and limited counts for some of the developmental stages/locations. The number of screened extracts for each location/developmental stage is shown between brackets. A full overview of all screening results can be found in Supplementary Table S1.

To get an indication of the abundance of these novel viruses in the Belgian tick population, we screened all 239 RNA pools by RT-PCR for the presence of the newly discovered viruses mentioned above, as well as the viruses recently discovered in *I. ricinus* from Norway (see Pettersson et al., 2017) and TBEV. An overview of the screening results is shown in Fig. 5, and a table detailing all PCR results is provided in Supplementary Table S1. It should be noted that differences in sample storage and sample processing might have affected the observed results and that quantitatively comparing the groups might not be meaningful, given the strong divergence in group sizes. Except for TBEV, all viruses were detected in one or more pools. Norway luteo-virus 4 was not detected by PCR, but some reads corresponding to this virus were present in one of the Illumina libraries. Presumably, the abundance of this virus was too low and/or the sample quality insufficient to allow detection by PCR. Especially Norway partiti-like virus 1 and Grotenhout virus were highly abundant, being present in >50 per cent of pools screened. In addition, Leuven phlebovirus, Chimay rhabdovirus, and Bronnoya virus were also present in a significant portion of samples (17–27 per cent of pools), while all other viruses were only present sporadically (<6 per cent).

## 4. Discussion

Several publications in recent years have provided insight into the diversity of viruses carried by ticks (Tokarz et al., 2014, 2018; Xia et al., 2015; Moutailler et al., 2016; Pereira et al., 2017; Pettersson et al., 2017; Harvey et al., 2019; Meng et al., 2019; Ohlendorf et al., 2019). Although many unknowns remain for most of these viruses in terms of host and geographical range as well as pathological relevance for human, animal, and plant health, the discovery of novel viruses is an important first step in expanding our knowledge on viral diversity and viral evolution. As ticks are known vectors of important viral zoonoses, mapping the diversity of viruses carried by ticks can also aid in identifying the origin of yet unidentified or emerging zoonoses. In this study, we report the discovery of several novel viruses found in *I. ricinus* by metagenomic sequencing. In addition, we screened >200 tick pools from different sampling locations in Belgium for the presence of these and other, previously described, viruses. While the differences in sample storage conditions, the number of samples per location, and the spread of the used sampling sites throughout Belgium do not allow unbiased quantification of the spread of these viruses in the Belgian tick population, the data presented here do provide an indication of virus prevalence. Some viruses are detected in up to 75 per cent of pools, while most are only found sporadically. We detected no TBEV, in line with previous studies screening for TBEV in Belgian ticks (Lernout et al., 2019). Interestingly, most viruses are found in both adult and nymph stages and in several locations. Absence of certain viruses in certain developmental stages/locations can be most likely attributed to under-sampling of those conditions for those specific viruses, although the sample set would need to be expanded significantly to prove this. However, it is interesting to note that several of the viruses we detected, especially the more abundant ones, are found in multiple developmental stages, indicating that they are likely to have ticks as their natural host. Their finding in larval and nymph stages also seems to suggest that these viruses are transmitted vertically rather than being mammalian viruses acquired during one of the blood meals. Furthermore, the hypothesis that some of these viruses are strictly tick-associated is supported by the finding of related virus species in other *Ixodidae* species. For example, the blacklegged tick (*I. scapularis*), a common hard-bodied tick species in the east of North America, has been reported to carry nairo-, phlebo-, and rhabdoviruses that are closely related to Grotenhout virus, Leuven phlebovirus, and Chimay rhabdovirus, respectively (see (Pettersson et al., 2017; Vanmechelen et al., 2017; and Fig. 2) (Tokarz et al., 2014, 2018). The discovery of related virus species in related tick species indicates a long history of virus-tick co-evolution. Comparable to Grotenhout virus and Leuven phlebovirus, M segments have not yet been discovered for the related nairo- and phleboviruses discovered in *I. scapularis*, further illustrating their shared evolutionary origin. How these viruses manage to persist without a glycoprotein-encoding M segment, whether infectious particles can still be formed, and whether they can also be spread to other organisms remain to be determined.

Perhaps, the most interesting discovery is the finding of two members of the genus *Coltivirus.* This genus currently contains five member species, although putative additional species have been reported (Attoui et al., 2002; Gao et al., 2020; Harvey et al., 2019; Walker et al., 2020). Two of its members, Colorado tick fever virus and Eyach virus, are known human pathogens capable of causing severe disease (Attoui et al., 2005). While the other members, Tarumizu tick virus, Tai Forest reovirus, and Kundal virus, have not been implicated in human disease, they have been shown to be capable of infecting mammalian and/or human cell lines (Fujita et al., 2017; Weiss et al., 2017; Yadav et al., 2019). One of the two coltiviruses reported here appears to be a strain of Eyach virus, sharing >94 per cent nucleotide similarity for all but one segments. To our knowledge, this marks the first detection of Eyach virus or any known viral human pathogen in the Belgian tick population, further expanding the known geographical range in which this virus is found (see Chastel 1998). Interestingly, the fourth segment is markedly dissimilar from known Eyach virus sequences, indicating that some form of reassortment has occurred, hinting at the existence of other, yet to be discovered coltiviruses. The fourth segment encodes for VP4, a protein that contains no known conserved domains and, while conserved among members of the genus *Coltivirus*, seems to share no similarities with any known proteins of other reoviruses. As such, evaluating the importance of this reassortment is difficult, although it is possible that the impact on the virus’ pathogenicity or other characteristics is severe, as even minor sequence variations have been observed to significantly affect coltivirus infections *in vitro* (Fujita et al., 2017). Unlike this strain of Eyach virus, Gierle tick virus clearly represents a novel species within the genus *Coltivirus*, with all 12 segments sharing <70 per cent nucleotide similarity with other known members of the genus. Although we did not manage to isolate the virus itself, its close phylogenetic relationship to other viruses all known to infect mammalian cells does warrant further research into this virus as a putative human or animal pathogen.

In addition to two coltiviruses, we also identified two reoviruses not belonging to any of the currently recognized genera in the family *Reoviridae*. While we only found three partial segments for one of them, Torhout tick virus appears to be distantly related to Fennes virus, a virus recently identified in ticks found on Antarctic penguins (Wille et al., 2020). Conversely, a near-complete genome was reconstructed for Zoersel tick virus. Phylogenetically, this virus is most closely related to a group of viruses for which only six genome segments are known, and, to a lesser extent, the ten-segment containing Operophtera brumata reovirus. Interestingly, several of the ten segments we identified for Zoersel tick virus share (almost) no sequence similarity with any known sequences. Without the minimal sequence similarity between segments 5, 7, and 10 and the corresponding segments of Operophtera brumata reovirus (NC_007559-NC_007568), identifying all ten segments would have been significantly more difficult. Thanks to the availability of this reference, the erroneous conclusion that Zoersel tick virus has only six genome segments could be avoided. The high degree of sequence divergence of the additional segments of Zoersel tick virus illustrates the likely possibility that its closest known relatives might also have additional segments that are highly divergent. Such divergence could leave certain segments undiscovered by typical BLAST-based approaches, a concern previously raised by some of the groups that discovered these putative six-segmented viruses (Sadeghi et al., 2017; Medd et al., 2018; Temmam et al., 2019). Re-analysing these viruses’ sequencing sets with the help of additional related sequences, such as the ones presented here, might help identifying potentially missed segments.

For several of the viruses we detected in our sequencing data, highly similar or related viruses have previously been reported in different tick species from various locations, strengthening the hypothesis that these viruses are truly tick-associated. As noted by Pettersson and colleagues, this association can sometimes be difficult to claim based only on sequencing data, especially if the viruses in question are only found infrequently or in low abundance (Pettersson et al., 2017). Intriguingly, two of the viruses for which they note such low abundance, Norway mononegavirus 1 and Norway luteo-virus 4, are also some of the least-detected viruses in our sample set, although we did find traces of both. Their detection in multiple unrelated datasets might suggest that they are simply low abundant tick-associated viruses, rather than environmental contaminants. In addition to previously identified viruses, we also detected a variety of novel viruses in our data. For some of these viruses, (near-)complete genomes could be reconstructed (see above), albeit most were only present in low abundance, resulting in only small fragments of their genomes being recovered. The low abundance and associated lack of available sequence data make it difficult to confidently classify these viruses as tick-associated, although the similarity of some of them with members of virus families and orders known to contain tick-borne viruses (*Rhabdoviridae*, *Phenuiviridae*, *Chuviridae*, *Orthomyxoviridae*, and *Mononegavirales*) do seem to support this association. For many other viruses of which we find traces, which only share similarities with unclassified viruses or viruses belonging to virus lineages with mixed hosts, the origin remains largely unknown. Nonetheless, they do provide further evidence that virus diversity in ticks (and other arthropods) is much higher than until recently known. Additional studies are needed to further map this diversity, allowing a better understanding of tick-associated virus evolution. A better characterization of the virus diversity in tick populations can also facilitate studying the spread of known and novel zoonotic viruses, allowing more targeted pre- and post-exposure countermeasures for tick-borne diseases to be taken where needed.

In conclusion, we present the first study looking at virus diversity in *I. ricinus* from Belgium. We show that Belgian ticks carry a wide variety of viruses and that several of these viruses are found in different locations and different tick developmental stages. We also managed to reconstruct (near-)complete genome sequences for several novel viruses, including a new strain of the pathogenic Eyach virus, a new coltivirus species, and a highly divergent reovirus that remains to be classified. This research further illustrates that many tick-associated viruses remain to be discovered and that additional studies are needed to expand our knowledge of virus diversity in ticks and to further characterize these novel viruses.

## Supplementary Material

veab066_Supp

## Data Availability

Virus sequences generated in this study have been submitted to GenBank under accession numbers MW874081-MW874117.

## References

[R1] Attoui H. et al. (2002) ‘Genus Coltivirus (Family Reoviridae): Genomic and Morphologic Characterization of Old World and New World Viruses’, *Archives of Virology*, 147: 533–61. 11958454 10.1007/s007050200005 PMC7098428

[R2] ——— et al. (2005) ‘Coltiviruses and Seadornaviruses in North America, Europe, and Asia’, *Emerging Infectious Diseases*, 11: 1673–9. 16318717 10.3201/eid1111.050868 PMC3367365

[R3] Boulanger N. et al. (2019) ‘Ticks and Tick-Borne Diseases’, *Médecine Et Maladies Infectieuses*, 49: 87–97. 30736991 10.1016/j.medmal.2019.01.007

[R4] Buchfink B., Xie C., and Huson D. H. (2015) ‘Fast and Sensitive Protein Alignment Using DIAMOND’, *Nature**Methods*, 12: 59–60. 10.1038/nmeth.3176 25402007

[R5] Bush L. M., and Vazquez-Pertejo M. T. (2018) ‘Tick Borne Illness-Lyme Disease’, *Disease-a-Month*, 64: 195–212. 29402399 10.1016/j.disamonth.2018.01.007

[R6] Capella-Gutierrez S., Silla-Martinez J. M., and Gabaldon T. (2009) ‘trimAl: A Tool for Automated Alignment Trimming in Large-Scale Phylogenetic Analyses’, *Bioinformatics*, 25: 1972–3. 19505945 10.1093/bioinformatics/btp348 PMC2712344

[R7] Chastel C. (1998) ‘Erve and Eyach: Two Viruses Isolated in France, Neuropathogenic for Man and Widely Distributed in Western Europe’, *Bulletin De l**’Academie Nationale De Medecine*, 182: 801–9; discussion 09–10. 9673063

[R8] Conceicao-Neto N. et al. (2018) ‘Netovir: Modular Approach to Customize Sample Preparation Procedures for Viral Metagenomics’, *Methods in Molecular Biology (Clifton, N.J.)*, 1838: 85–95. 10.1007/978-1-4939-8682-8_7 30128991

[R9] Fujita R. et al. (2017) ‘Isolation and Characterization of Tarumizu Tick Virus: A New Coltivirus from Haemaphysalis Flava Ticks in Japan’, *Virus**Research*, 242: 131–40. 10.1016/j.virusres.2017.09.017 28964878

[R10] Gao W. H. et al. (2020) ‘Newly Identified Viral Genomes in Pangolins with Fatal Disease’, *Virus**Evolution*, 6: veaa020. 10.1093/ve/veaa020 PMC7151644 32296543

[R11] Graham R. I. et al. (2006) ‘Detection and Characterisation of Three Novel Species of Reovirus (Reoviridae), Isolated from Geographically Separate Populations of the Winter Moth Operophtera Brumata (Lepidoptera: Geometridae) on Orkney’, *Journal of Invertebrate Pathology*, 91: 79–87. 16413573 10.1016/j.jip.2005.11.003

[R12] Guglielmone A. A. et al. (2010) ‘The Argasidae, Ixodidae and Nuttalliellidae (Acari: Ixodida) of the World: A List of Valid Species Names’, *Zootaxa*, 2528: 1–28.

[R13] Harvey E. et al. (2019) ‘Extensive Diversity of RNA Viruses in Australian Ticks’, *Journal of Virology*, 93: 3. 10.1128/JVI.01358-18 PMC6340049 30404810

[R14] Hubalek Z., and Rudolf I. (2012) ‘Tick-borne Viruses in Europe’, *Parasitology Research*, 111: 9–36. 22526290 10.1007/s00436-012-2910-1

[R15] Katoh K., and Standley D. M. (2013) ‘MAFFT Multiple Sequence Alignment Software Version 7: Improvements in Performance and Usability’, *Molecular Biology and Evolution*, 30: 772–80. 23329690 10.1093/molbev/mst010 PMC3603318

[R16] Koren S. et al. (2017) ‘Canu: Scalable and Accurate Long-read Assembly via Adaptive k Mer Weighting and Repeat Separation’, *Genome**Research*, 27: 722–36. 10.1101/gr.215087.116 PMC5411767 28298431

[R17] Kuivanen S. et al. (2019) ‘Detection of Novel Tick-borne Pathogen, Alongshan Virus, in Ixodes Ricinus Ticks, South-eastern Finland, 2019’, *Eurosurveillance*, 24: 27. 10.2807/1560-7917.ES.2019.24.27.1900394 PMC6628756 31290392

[R18] Lernout T. et al. (2019) ‘Prevalence of Pathogens in Ticks Collected from Humans through Citizen Science in Belgium’, *Parasites and Vectors*, 12: 550. 10.1186/s13071-019-3806-z PMC6873681 31752967

[R19] Li C. X. et al. (2015) ‘Unprecedented Genomic Diversity of RNA Viruses in Arthropods Reveals the Ancestry of Negative-sense RNA Viruses’, *Elife*, 4: e05378. 10.7554/eLife.05378 PMC4384744 25633976

[R20] Madison-Antenucci S. et al. (2020) ‘Emerging Tick-Borne Diseases’, *Clinical Microbiology Reviews*, 33: 2. 10.1128/CMR.00083-18 PMC6941843 31896541

[R21] Mansfield K. L. et al. (2017) ‘Emerging Tick-Borne Viruses in the Twenty-First Century’, *Frontiers in Cellular and Infection Microbiology*, 7: 298. 10.3389/fcimb.2017.00298 PMC5504652 28744449

[R22] Medd N. C. et al. (2018) ‘The Virome of Drosophila Suzukii, an Invasive Pest of Soft Fruit’, *Virus**Evolution*, 4: vey009. 10.1093/ve/vey009 PMC5888908 29644097

[R23] Meng F. et al. (2019) ‘Virome Analysis of Tick-borne Viruses in Heilongjiang Province, China’, *Ticks**and Tick-borne Diseases*, 10: 412–20. 10.1016/j.ttbdis.2018.12.002 30583876

[R24] Moutailler S. et al. (2016) ‘Diversity of Viruses in Ixodes Ricinus, and Characterization of a Neurotropic Strain of Eyach Virus’, *New Microbes and**New**Infections*, 11: 71–81. 10.1016/j.nmni.2016.02.012 PMC4845080 27158509

[R25] Nguyen L. T. et al. (2015) ‘IQ-TREE: A Fast and Effective Stochastic Algorithm for Estimating Maximum-likelihood Phylogenies’, *Molecular Biology and Evolution*, 32: 268–74. 25371430 10.1093/molbev/msu300 PMC4271533

[R26] Obsomer V. et al. (2013) ‘Spatial Disaggregation of Tick Occurrence and Ecology at a Local Scale as a Preliminary Step for Spatial Surveillance of Tick-borne Diseases: General Framework and Health Implications in Belgium’, *Parasites and Vectors*, 6: 190. 10.1186/1756-3305-6-190 PMC3726513 23800283

[R27] Ohlendorf V. et al. (2019) ‘Huge Diversity of Phleboviruses in Ticks from Strandja Nature Park, Bulgaria’, *Ticks**and Tick-borne Diseases*, 10: 697–703. 10.1016/j.ttbdis.2019.03.001 30871930

[R28] Ondov B. D., Bergman N. H., and Phillippy A. M. (2011) ‘Interactive Metagenomic Visualization in a Web Browser’, *BMC Bioinformatics*, 12: 385. 10.1186/1471-2105-12-385 PMC3190407 21961884

[R29] Pereira A. et al. (2017) ‘Multiple Phlebovirus (Bunyaviridae) Genetic Groups Detected in Rhipicephalus, Hyalomma and Dermacentor Ticks from Southern Portugal’, *Ticks**and Tick-borne Diseases*, 8: 45–52. 10.1016/j.ttbdis.2016.09.015 27717757

[R30] Pettersson J. H. et al. (2017) ‘Characterizing the Virome of Ixodes Ricinus Ticks from Northern Europe’, *Scientific Reports*, 7: 10870. 10.1038/s41598-017-11439-y PMC5589870 28883464

[R31] Potter S. C. et al. (2018) ‘HMMER Web Server: 2018 Update’, *Nucleic Acids**Research*, 46: W200–4. 10.1093/nar/gky448 PMC6030962 29905871

[R32] Sadeghi M. et al. (2017) ‘Genomes of Viral Isolates Derived from Different Mosquitos Species’, *Virus**Research*, 242: 49–57. 10.1016/j.virusres.2017.08.012 PMC5665172 28855097

[R33] Shen W. et al. (2016) ‘Seqkit: A Cross-Platform and Ultrafast Toolkit for FASTA/Q File Manipulation’, *PLoS One*, 11: e0163962. 10.1371/journal.pone.0163962 PMC5051824 27706213

[R34] Shi J. et al. (2018) ‘Tick-Borne Viruses’, *Virologica Sinica*, 33: 21–43. 29536246 10.1007/s12250-018-0019-0 PMC5866268

[R35] Shi M. et al. (2016) ‘Redefining the Invertebrate RNA Virosphere’, *Nature*, 540: 539–43. 27880757 10.1038/nature20167

[R36] Temmam S. et al. (2019) ‘Six Nearly Complete Genome Segments of a Novel Reovirus Identified in Laotian Batflies’, *Microbiology Resource Announcements*, 8: 26. 10.1128/MRA.00733-19 PMC6856264 31727698

[R37] Tokarz R. et al. (2014) ‘Virome Analysis of Amblyomma Americanum, Dermacentor Variabilis, and Ixodes Scapularis Ticks Reveals Novel Highly Divergent Vertebrate and Invertebrate Viruses’, *Journal of Virology*, 88: 11480–92. 25056893 10.1128/JVI.01858-14 PMC4178814

[R38] ——— et al. (2018) ‘Identification of Novel Viruses in Amblyomma Americanum, Dermacentor Variabilis, and Ixodes Scapularis Ticks’, *Msphere*, 3: e00614–17. 10.1128/mSphere.00614-17 PMC5853492 29564401

[R39] Vanmechelen B. et al. (2017) ‘Grotenhout Virus, a Novel Nairovirus Found in Ixodes Ricinus in Belgium’, *Genome**Announcements*, 5: 21. 10.1128/genomeA.00288-17 PMC5477388 28546475

[R40] Walker M. (2019) ‘The Biology and Ecology of the Sheep Tick Ixodes Ricinus’.

[R41] Walker P. J. et al. (2020) ‘Changes to Virus Taxonomy and the Statutes Ratified by the International Committee on Taxonomy of Viruses (2020)’, *Archives of Virology*, 165: 2737–48. 32816125 10.1007/s00705-020-04752-x

[R42] Weiss S. et al. (2017) ‘A Novel Coltivirus-related Virus Isolated from Free-tailed Bats from Cote d’Ivoire is Able to Infect Human Cells in Vitro’, *Virology Journal*, 14: 181. 10.1186/s12985-017-0843-0 PMC5604424 28923111

[R43] Wille M. et al. (2020) ‘Sustained RNA Virome Diversity in Antarctic Penguins and their Ticks’, *The**ISME Journal*, 14: 1768–82. 10.1038/s41396-020-0643-1 PMC7305176 32286545

[R44] Xia H. et al. (2015) ‘Metagenomic Profile of the Viral Communities in Rhipicephalus Spp. Ticks from Yunnan, China’, *PLoS One*, 10: e0121609. 10.1371/journal.pone.0121609 PMC4370414 25799057

[R45] Yadav P. D. et al. (2019) ‘Characterization of Novel Reoviruses Wad Medani Virus (Orbivirus) and Kundal Virus (Coltivirus) Collected from Hyalomma Anatolicum Ticks in India during Surveillance for Crimean Congo Hemorrhagic Fever’, *Journal of Virology*, 93: e00106–19. 10.1128/JVI.00106-19 PMC6580951 30971476

